# Haemotrophic Mycoplasmas Infecting Pigs: A Review of the Current Knowledge

**DOI:** 10.3390/microorganisms12071267

**Published:** 2024-06-22

**Authors:** Julia Ade, Matthias Eddicks, Mathias Ritzmann, Katharina Hoelzle, Ludwig E. Hoelzle, Julia Stadler

**Affiliations:** 1 Department of Livestock Infectiology and Environmental Hygiene, Institute of Animal Science, University of Hohenheim, 70593 Stuttgart, Germany; 2 Clinic for Swine, Centre for Clinical Veterinary Medicine, Ludwig-Maximilians-Universität München, 85764 Oberschleissheim, Germany

**Keywords:** haemoplasmas, *Mycoplasma suis*, *Mycoplasma parvum*, ‘*Candidatus* Mycoplasma haemosuis’, swine anaemia

## Abstract

Haemotrophic mycoplasmas (haemoplasmas) are a group of highly specific and adapted bacteria. Three different haemoplasma species in pigs are known to date: *Mycoplasma* (*M*.) *suis*, *M. parvum* and ‘*Candidatus* (*Ca*.) M. haemosuis’. Even though these bacteria have been known in pig farming for a long time, it is difficult to draw general conclusions about the relevance of their infections in pigs. This review summarizes the current knowledge on the three porcine haemoplasma species with regards to clinical and pathological descriptions, pathobiology, epidemiology and diagnostics as well as prevention and therapy. Overall, it is clear that considerably more data are available for *M. suis* than for the other two species, but generally, porcine haemoplasmas were found to be highly prevalent all over the world. *Mycoplasma suis* is the most virulent species, causing acute infectious anaemia in pigs (IAP), whereas *M. parvum* usually results in chronic and subclinical infections associated with performance losses. Little is known about the clinical significance of the recently discovered third porcine species ‘*Ca*. M. haemosuis’. So far, the described pathogenic mechanisms mainly include direct destruction of erythrocytes via adhesion, invasion, eryptosis and nutrient scavenging, indirect erythrocyte lysis due to immune-mediated events and immune dysregulation processes. A review of published diagnostic data confirms PCR assays as the current standard method, with various cross-species and species-specific protocols. Overall, there is a need for further examination to obtain valuable insights for practical application, specifically regarding the importance of subclinical infections in naturally infected animals. An essential requirement for this will be to gain a more comprehensive understanding of the mechanisms operating between the host and the pathogen.

## 1. Introduction

Haemotrophic mycoplasmas (haemoplasmas) are a group of cell-wall-less, small and pleomorphic bacteria occurring in the blood of their mammal hosts [1]. They attach to or invade erythrocytes, whereas parasitism outside of the blood has not yet been discovered [1,2,3]. Haemoplasmas are defined as highly adapted to the blood environment and strictly depend on their hosts, especially considering that the degradation of glucose seems to be the only complete pathway for self-sufficient energy metabolism [1,4]. Despite the fact that haemoplasmas have been known for almost 100 years, they still cannot be cultivated in vitro, which presents a major challenge in gaining knowledge on the pathobiology of these organisms as well as in the establishment of therapeutic and preventive measures [2,5,6]. Several haemoplasma species are described in various mammal hosts, including farm animals, wild animals and humans [1,7]. The taxonomy of haemoplasmas has been revised in the past and is still not yet completed. Haemoplasmas were initially differentiated into two different genera–*Eperythrozoon* and *Haemobartonella*. Based on sequence analyses of the 16S rRNA and RNase P genes, the genera were reclassified from the order *Rickettsiales*, family *Anaplasmataceae*, into the order *Mycoplasmatales*, family *Mycoplasmataceae,* genus *Mycoplasma* [8,9,10,11,12,13]. Phylogenetic analysis revealed a clear separation of the haemoplasmas from the other mycoplasma species into their own cluster. Within this cluster, demarcation of two subgroups can be seen, with one of the subgroups containing the haemoplasma species formerly assigned to the genus *Eperythrozoon* (Suis group) and the other subgroup (Haemofelis group) containing the representatives of the former genus *Haemobartonella* [13,14,15,16]. In 2018, a revision of the taxonomy, with the creation of a new genus, was proposed by Gupta et al.; however, this proposal has so far not been implemented [17]. The genome of haemoplasmas is quite small, harbouring between 547 and 1545 coding sequences (CDSs) [18]. A high percentage of the CDSs code for hypothetical proteins [18]. Analyses revealed major gaps in metabolic pathways, suggesting a very close adaption of haemoplasmas to their host environment [18].

Pigs are a well-known host and infections caused by porcine haemoplasma species are often of major relevance in the porcine health management and pig production sectors [19,20,21,22,23,24]. This review aims to summarize the current knowledge on haemoplasma infections in pigs, embracing the current taxonomy, clinical and pathological aspects as well as epidemiological descriptions and diagnostics.

## 2. Porcine Haemoplasma Species

To date, numerous haemoplasma species related to those that infect porcine have been described, with the first haemoplasma in pigs described in the 1930s [25,26]. The first species description was at the beginning of the 1950s when Splitter differentiated the two species *Eperythrozoon* (*E.*) *suis* and *Eperythrozoon parvum* [27,28], which are currently known as *Mycoplasma* (*M.*) *suis* and *Mycoplasma parvum* [9,10,29]. In 2017, a third haemoplasma species named ‘*Candidatus* (*Ca*.) Mycoplasma haemosuis’ was described in China [30]. Recently, Thongmeesee et al. have described the existence of a putative novel porcine haemoplasma species in Thailand [15]. Table 1 below lists the currently confirmed porcine haemoplasma species. In the phylogenetic cluster, *M. suis*, *M. parvum* and the putative novel species all belong to the “Suis group”, while ‘*Ca*. M. haemosuis’ is in the “Haemofelis group” [15,31].

Complete genome sequences are available for *M. suis* and *M. parvum*, but not for ‘*Ca*. M. haemosuis’ [4,29,31,32]. Like other haemoplasmas, the genomes of both porcine species harbour a high percentage of CDSs with unknown function (hypothetical proteins), with 61.3% hypothetical proteins in *M. suis* strain Illinois [4], 64.6% in *M. suis* strain KI3806 [32] and 77% in *M. parvum* strain Indiana [29]. Interestingly, a comparison between *M. suis* strain Illinois and *M. parvum* strain Indiana showed that both species share all CDSs with known functions [33]. To gain more insights into the pathobiology of haemoplasmas as well as to understand differences in the pathogenicity of different species, haemoplasma hypothetical proteins should be a topic of further research. Further, full-genome analysis of the novel ‘*Ca*. M. haemosuis’ is essential.

## 3. Clinical and Pathological Descriptions

The following section provides an overview of published data describing the clinical and pathological pictures of individual porcine haemoplasmas. Available literature for *M. suis* is considerably more extensive than for the other two species. It can be summarized that porcine haemoplasmas are associated with acute and clinically apparent diseases, as well as with subclinical and most likely chronic diseases. All forms of diseases can contribute to economic losses in pig production [20,21,22,34].

Considering the acute form of the disease, *M. suis* is the causative infectious agent of infectious anaemia in pigs (IAP), a disease formerly known as porcine eperythrozoonosis (PE). ‘*Ca*. M. haemosuis’ was associated with IAP in two published cases but confirmation of pathogenicity in experimental infection is still pending [30,35]. Studies of *M. parvum* are not extensive and are very rare and old, leading to uncertainty about the capacity of *M. parvum* to cause acute IAP.

Data on the chronic form of infection are scarce, but implicate a negative impact of *M. suis*, and possibly *M. parvum*, on the performance and health of pigs. So far, no data has been published on the chronic form of ‘*Ca*. M. haemosuis’ infection. As presented in Chapter 5.2., the detection of the haemoplasma species in animals without obvious clinical signs indicates a widespread occurrence of subclinical or asymptomatic forms of the disease.

Co-infections with two or all three haemoplasma species have been described in subclinical animals in the field in a couple of cases [36,37,38,39]; however, the relevance of those co-infections has not been discussed in detail.

### 3.1. Mycoplasma suis

Clinical signs of *M. suis* infections have been comprehensively described. Experimental infections with *M. suis* in splenectomised pigs imitate the acute and severe form of IAP, as the absence of the spleen probably results in insufficient removal of *M. suis*-infected erythrocytes from the blood [3,40]. The cardinal signs of acute IAP are pallor and icterus of the skin and mucous membranes, both resulting from intravascular haemolysis [3,40,41,42,43,44]. Further, skin petechia or urticaria as well as necrosis and cyanoses of distal body parts, particularly of the ears, distal limbs and the tail can be observed [3,45]. Generalized haemorrhages of the skin are usually referred to as *Morbus maculosus* or purpura haemorrhagica [45]. In addition, unspecific signs of infections like high fever, apathy and an increased respiratory rate also occur [3,40,42,45]. In sows, dysgalactia, reproductive cycle disorders and abortion are described in the literature [23,43,46]. Laboratory findings of acute IAP are typically represented by a high number of *M. suis* cells in the blood (up to >10^10^/mL), as well as by normocytic, normochromic anaemia, increased bilirubin levels and hypoglycaemia [3,40,45,47,48]. Acute IAP is a life-threatening condition that can be fatal, especially in piglets, if no treatment is initiated [3,40]. Outbreaks of acute and severe IAP in the field seem to be rare and are attributed to certain stressors like weaning, parturition or re-grouping [49,50]. The severity of acute IAP in the field varies between individuals and different age groups, with younger animals usually developing more severe symptoms, whereas older animals often exhibit mild symptoms and may transition to the chronic form of the disease. Chronic infections are often subclinical/asymptomatic, though they can cause low-grade anaemia. Chronic infections are usually characterized by a much smaller number of pathogens in the blood compared to acute IAP [40,51]. Many authors consider infections with *M. suis* to be life-long [40,50].

Interestingly, *M. suis* infections appear to predispose the host to enteric and respiratory diseases, as described in older studies [52,53]. Pereyra et al. further stated a potential connection between *M. suis* infections and “postweaning multisystemic wasting syndrome” (PMWS), a condition associated with porcine circovirus type 2 (PCV 2) infection [54]. In addition, a negative impact of *M. suis* infections on performance parameters has been outlined. studies by Petri et al. and Sonalio et al. observed a negative correlation between the occurrence of haemoplasmas, including *M. suis,* and daily weight gain, slaughter weights and the number of weaned piglets [22,34]. Zinn et al. described reduced birth weights and reduced weights in 3-week-old piglets but no further effects on reproductive performance in *M. suis*-positive farms [24]. A more recent study showed significantly increased stillborn rates per litter in farms where *M. suis* was detected compared to *M. suis*-negative farms, but no significant changes concerning other reproductive parameters (i.e., number of live born piglets per sow, number of weaned piglets per sow or return to oestrus rate) [20]. Similarly, the study by Brissonnier et al. confirmed an increased rate of stillborn piglets in *M. suis*-infected gilts; however, significant negative impacts of *M. suis* on other production performance parameters were not obvious in sows [21].

**Figure 1:**
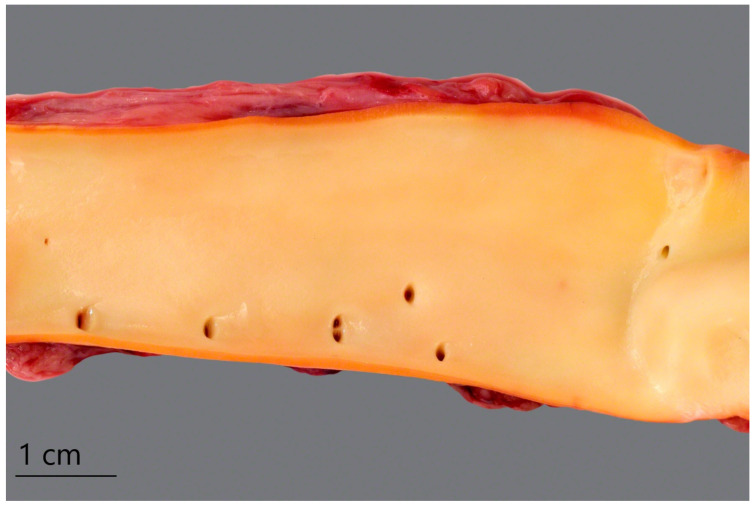
Yellowish-icteric discolouration of the tunica intima of an aorta obtained from an *M. suis*-infected pig suffering from acute IAP [3] (published open access under a Creative Commons Attribution 4.0 International License: https://creativecommons.org/licenses/by/4.0/).

The most frequently described pathomorphological findings in pigs who suffer from acute *M. suis*-induced IAP reflect the clinically observed icterus in various locations, such as the tunica intima of vessels [3,42,55,56]. As an example, Figure 1 illustrates the yellow discolouration of an aorta obtained from a pig infected with *M. suis* and suffering from acute IAP [3].

Macroscopic and microscopic evidence of disseminated intravascular coagulopathy, including hyaline vascular thrombi in organs and fibrin within lymph node sinuses, have been described [3]. Damage to the endothelial surface has also been described [57]. Table 2 provides a detailed summary of pathomorphological findings in pigs suffering from *M. suis*-induced IAP.

### 3.2. Mycoplasma parvum

A few studies on the pathogenicity of *M. parvum* have been published. According to these studies, this haemoplasma species does not cause severe infectious anaemia and is even considered non-pathogenic in pigs [33].

These studies were carried out on naturally infected pigs that were then splenectomised. Subsequently, bacteraemia is exaggerated, resulting in different clinical observations [27,33,60,61]. While two of these studies did not reveal any clinical signs in the infected pigs (age and usage unknown) following splenectomy [27,60], Jennings and Seamer reported that one out of seven infected animals (pigs less than 3 months old) developed mild anaemia and fever [61]. Further, in the most recent study conducted in 2014, the one animal utilized (a pig 6 months of age) exhibited a slight increase in body temperature during the peak of bacteraemia infection (10^10^ *M. parvum* cells/mL blood). However, while the packed red cell volume of this animal varied over the course of the study, it only dropped slightly below the low end of the normal reference range at one time point [33]. Experimental infection with *M. parvum* in negative weaned piglets resulted in clinically apparent disease with severe anaemia in splenectomised animals, whereas non-splenectomised animals remained clinically unremarkable [62]. According to extensive literature research, no further experimental infections with *M. parvum* have been conducted to date. Therefore, the assumption that this haemoplasma species does not typically cause acute IAP should be interpreted with caution and future studies should consider including more animals and different strains of *M. parvum*.

During field studies in pigs without obvious clinical deviations, *M. parvum* was frequently detected with mean bacterial blood loads similar to those found in subclinical *M. suis* field infections [36,37]. Interestingly, two studies examining the correlation between the presence of haemoplasmas and lower daily weight gains and lower slaughter weights in otherwise healthy fattening pigs, as well as lower numbers of weaned piglets in otherwise healthy sows, identified *M. parvum* as the dominant species among the diagnosed haemoplasmas [22,34].

Reports of macroscopic or microscopic findings in *M. parvum*-infected pigs have not been published to date.

### 3.3. ‘Candidatus Mycoplasma haemosuis’

**Figure 2:**
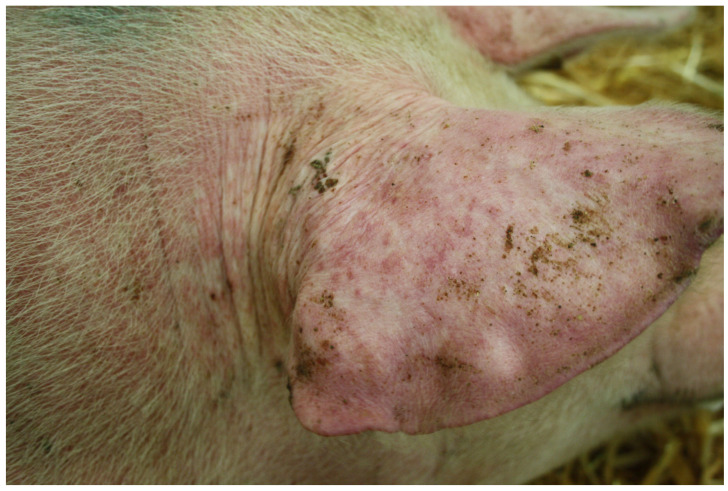
Cutaneous haemorrhages on the ear and ear base of a pig infected with ‘*Candidatus* Mycoplasma haemosuis’ [35] (figure owned by the authors, license for re-use is granted by John Wiley and Sons, License Number 5810131448483).

Like *M. suis*, ‘*Ca*. M. haemosuis’ is reported to cause infectious anaemia in pigs, as outlined in two publications [30,35]. Fu et al. mentioned one single piglet showing signs of infectious anaemia associated with a ‘*Ca*. M. haemosuis’-positive PCR result; however, clinical signs were not described further [30]. Stadler et al. described an acute disease event in fattening pigs caused by ‘*Ca*. M. haemosuis’. In this case, 30% of a pig herd of 1200 animals showed various clinical signs typically associated with acute IAP. Signs included fever, apathy, pallor, icterus, cyanoses of the ears and legs as well as generalized skin reactions. Figure 2 shows the cutaneous haemorrhages on the ear of one of the diseased pigs [35].

Laboratory investigations further showed normocytic-normochromic anaemia in pigs infected with ‘*Ca*. M. haemosuis’ having bacterial loads of up to 3.7 × 10^7^ cells/mL blood in the absence of *M. suis* in the blood of affected pigs [35]. Validation of the pathogenic potential of ‘*Ca*. M. haemosuis’ via experimental infections are still needed. Postmortem examination was carried out in one of the acutely diseased pigs from the above-mentioned case report by Stadler et al. [35]. Macroscopic and microscopic investigations revealed icterus of the liver and vessels, pallor of mucus membranes, generalized cutaneous haemorrhages, ascites, splenic sinus hyperplasia, follicular hyperplasia and multifocal lymphoplasmacytic dermatitis [35].

Although identified in association with disease, ‘Ca. M. haemosuis’ infections have also been reported in clinically healthy pigs of different age groups [15,30,38]. As with *M. suis* and *M. parvum* infections, reported blood bacterial loads in subclinically infected pigs are lower than in those associated with acute disease events [35]. Whether subclinical infections with ‘*Ca*. M. haemosuis’ have similar production impact as that described for *M. suis* has yet to be investigated.

## 4. Pathobiology and Host–Pathogen Interactions

Knowledge of host–pathogen interactions between haemoplasmas and the porcine host is incomplete, and the acquisition of new insights is hampered by the inability to culture porcine haemoplasmas in vitro. Published information on the pathobiology of porcine haemoplasmas and host–pathogen interactions are limited to *M. suis*, as described in this section.

Pathobiological aspects of *M. suis* were comprehensively reviewed by Hoelzle et al. in 2014 [2]. In *M. suis*-infected animals, the cardinal sign of haemolytic anaemia is direct and indirect erythrocyte destruction. Direct ways include erythrocytic destruction induced by cell death, referred to as eryptosis, and extravascular phagocytosis [2]. Both are results of adhesion and invasion of *M. suis* on and into RBCs [44,63,64,65,66] as well as probable nutrient scavenging [4,48,67,68,69]. Indirect destruction of RBCs can be a result of *M. suis*-induced auto-reactive IgM and IgG antibodies [70,71,72,73]. At the molecular level, several *M. suis* proteins have been investigated that appear to be involved in adhesion [65,74,75,76,77]. Interestingly, some of these proteins also function in glucose [65,74]. Table 3 lists these proteins. For MSG1, α-Enolase and OSGEP, the adhesion to erythrocytes has been confirmed in in vitro trials [65,74,75]; However, the function of HspA1 and the seven hypothetical *M. suis* proteins identified in the study by Dietz et al. as adhesion proteins is only suspected [76,77]. Furthermore, MSG1 and α-Enolase are known to also have enzymatic functions in the degradation of glucose.

Erythrocyte destruction is clearly associated with the described clinical signs of icteroanaemia. In addition, it is also conceivable that low-grade but persistent erythrocytic destruction and mild anaemia in chronically infected animals can lead to increased susceptibility to respiratory and enteric diseases described for *M. suis*, as well as to the performance losses described for *M. suis* and *M. parvum*.

Apart from interaction and damage to erythrocytes, targeting of endothelial cells resulting in endothelial damage was demonstrated in an in vitro study [57]. Damage to the endothelium likely contributes to the development of coagulation disorders, including disseminated intravascular coagulation (DIC), frequently observed in acute *M. suis* IAP [3,78]. Additional factors that may lead to coagulopathies include circulating antigen–(auto-) antibody complexes, changes in the erythrocytic membranes or increased eryptosis [3,66]. Alterations in the coagulation system have also been observed during blood transcriptome analyses in an experimental pig model of chronic IAP [79].

In addition to autoantibody erythrocyte destruction, additional immunopathological mechanisms of host immune system modulation are also discussed. During acute IAP in splenectomised pigs, Zachary and Smith were able to observe a suppression in T-lymphocyte blastogenic responses [72]. A more recent study conducted by do Nascimento et al. demonstrated significant alterations in the blood transcriptome of pigs that were chronically infected with *M. suis*, including alterations in genes associated with immune functions [79]. Downregulation of genes associated with innate immunity, like tlr8, chemokines and chemokine receptors, suggests a general immune suppression in infected pigs [79]. This observation could, in turn, be associated with the described higher susceptibility of *M. suis*-infected animals to respiratory and enteric diseases and decreased production performance.

## 5. Epidemiology

### 5.1. Worldwide Occurrence

A literature search revealed evidence of the occurrence of porcine haemoplasmas in domestic pigs on all the continents. Most of the published data are based on individual case reports rather than on prevalence studies. Table 4 provides an overview of the available literature, describing the occurrence of porcine haemoplasmas in domestic pigs in different countries. It should be mentioned that most of the reports do not include a valid differentiation between the different haemoplasma species. *M. parvum* was definitively identified in only a couple of cases [15,22,27,29,30,33,34,36,39,60,61,62,80,81,82,83] and the novel ‘*Ca*. M. haemosuis’ has so far only been described in China, Korea, Thailand and Germany [15,30,35,38,82].

Besides their occurrence in domesticated pigs, *M. suis* and *M. parvum* have been detected in wild boars in Brazil (both haemoplasma species) and Germany (*M. suis* only) [116,117,118,119]. *M. suis* has also been described in white-lipped peccaries (*Tayassu pecar*) in Brazil [118].

### 5.2. Prevalence

Table 5 provides a summary of prevalence data from published studies that used molecular diagnostics to specifically identify the presence of the three known porcine haemoplasma species in domestic pigs. Only studies that sampled animals from more than one farm are included in this summary.

None of the listed publications reported clinical signs in animals included in these prevalence studies. Instead, the majority either claim the sampling of healthy animals, or no information is given on the clinical status of animals, giving the impression that obvious clinical signs of haemoplasma-related disease were not present at the time of sample collection. Summarizing the presented data, the three porcine haemoplasma species seem to be widely distributed, with prevalences of up to 72.2%. Comparative analyses of the prevalences of the different haemoplasma species are difficult to obtain from the data presented. The overall prevalence of *M. suis* appears to be higher than those of *M. parvum* and ‘*Ca*. M. haemosuis’; however, more data were collected for *M. suis*, which can lead to a distorted representation. Only two out of the cited studies, one from Korea [82] and one from Germany [37], allow a direct comparison of the three porcine haemoplasma species in the same samples. In both studies, *M. parvum* was detected more frequently than *M. suis* and ‘*Ca*. M. haemosuis’. Similarly, the study by Watanabe et al., which detected *M. suis* and *M. parvum*, describes more frequent detection of *M. parvum* compared to *M. suis* [36]. A possible explanation could be the lower virulence of *M. parvum* compared to *M. suis* and ‘*Ca*. M. haemosuis’, which may allow *M. parvum* to circulate undetected in the pig population for a longer time, providing more opportunity to spread between animals. Further studies are needed to confirm the predominance of *M. parvum* compared to *M. suis* and ‘*Ca*. M. haemosuis’ in the field. Regarding the different age groups, prevalences in fattening pigs seem to be more similar than in sows. Any interpretation regarding piglets and boars is not meaningful due to the different quantities of available data. The same is true for comparisons between regions or continents. Further trials should not only focus on the comparison of the different haemoplasma species but should also enable profound comparisons between age groups and geographic regions.

### 5.3. Transmission Routes

In general, haemoplasmas are known to be blood-transmitted bacteria. In the case of pigs, this can include ranking fights in groups iatrogenic transmission through reusing needles and zootechnical measures (i.e., castration, tail docking) [120]. Transmission of porcine haemoplasmas through blood-sucking arthropods has been proven in a few cases in the literature. In detail, Seamer verified the successful transmission of *M. parvum* via the pig louse (*Haematopinus suis*), Prullage described the successful transmission of *M. suis* via the stable fly (*Stomoxys calcitrans*) and the yellow fever mosquito (*Aedes aegypti*) under experimental conditions [62,121]. Further, *M. suis* and *M. parvum* have been detected in different arthropods in the field, like the pig louse (*Haematopinus suis*)and the stable fly (*Stomoxys calcitrans*) as well as in two different tick species (*Ambylomma sculptum* and *Ambylomma ovale*) [93,102,117].

Among the porcine haemoplasma species, studies on transmission routes have mainly been conducted with *M. suis* rather than with *M. parvum* and ‘*Ca*. M. haemosuis’. In experimental infections, *M. suis* and *M. parvum* are usually successfully transmitted via parenteral inoculation of *M. suis*/*M. parvum*-containing blood [3,33,40]. Oral transmission with *M. suis*-containing blood was successful using an inoculum with a very high bacterial load but unsuccessful with a lower-concentration inoculum representing quantities of *M. suis* that are typically found in field infections [120,122]. Therefore, oral transmission of *M. suis*-containing blood does not seem to play a role in field conditions [122].

Transmission of *M. suis* via artificial insemination of blood-contaminated semen to sows has also been described in the literature [123].

In addition to horizontal transmission, vertical transmission of *M. suis* and ‘*Ca*. M. haemosuis’ from sows to their offspring has been described [20,38,42,124].

Blood-independent shedding of *M. suis* has been demonstrated under experimental conditions, where *M. suis* DNA was detected in saliva, urine as well as in nasal and vaginal secretions during acute disease events of infectious anaemia [125].

On the contrary, another study failed to show the presence of *M. suis* and ‘*Ca*. M. haemosuis’ in urine, saliva and semen samples of naturally infected pigs in the field [122]. The authors therefore attribute a minor role to blood-independent transmission of porcine haemoplasmas [122].

## 6. Diagnostics

### 6.1. Cultivation

Any attempts to cultivate porcine haemoplasmas in vitro have failed so far and animal trials remain the only possible means of replicating these bacteria. Cultivation trials using *M. suis* resulted in the maintenance of bacterial numbers and transformation of bacterial cells to nanoforms but failed to replicate the organisms [6,69]. Those trials were conducted in porcine erythrocyte cultures [69] and in cell-free *Mycoplasma* media [6]. An obvious explanation for the unsuccessful cultivation attempts is the use of non-optimal media conditions. Haemoplasmas seem to be highly specialized and adapted to an environment that has not yet been sufficiently replicated [2].

### 6.2. Microscopy

**Figure 3:**
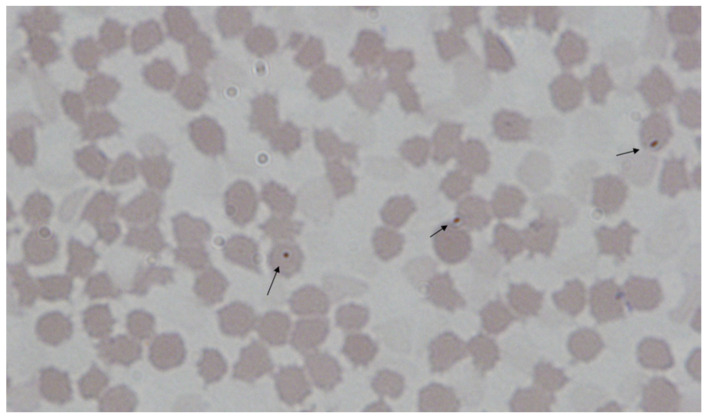
Giemsa-stained blood smear of a pig infected with ‘*Ca*. M. haemosuis’. ‘*Ca*. M. haemosuis’ cells occur as small purple dots on the red blood cells (indicated by arrows) [30] (published open access under the terms of the Creative Commons Attribution Non-Commercial No Derivatives (by-nc-nd) License: CC-BY-NC-ND 4.0: https://creativecommons.org/licenses/by-nc-nd/4.0/).

Due to the inability to cultivate porcine haemoplasmas, microscopic examination of blood smears was the standard method for diagnosing haemoplasmosis in the pre-PCR era. Light microscopy with Romanowsky staining methods like Giemsa or Wright has been used traditionally [63,105,126]. Depending on the stain type and pH, haemoplasmas appear as individual or clustered pale red to reddish-purple dots on the surface of erythrocytes or free in the plasma [59,63,64,105,126]. Figure 3 shows a Giemsa-stained blood smear of a pig infected with ‘*Ca*. M. haemosuis’.

**Figure 4:**
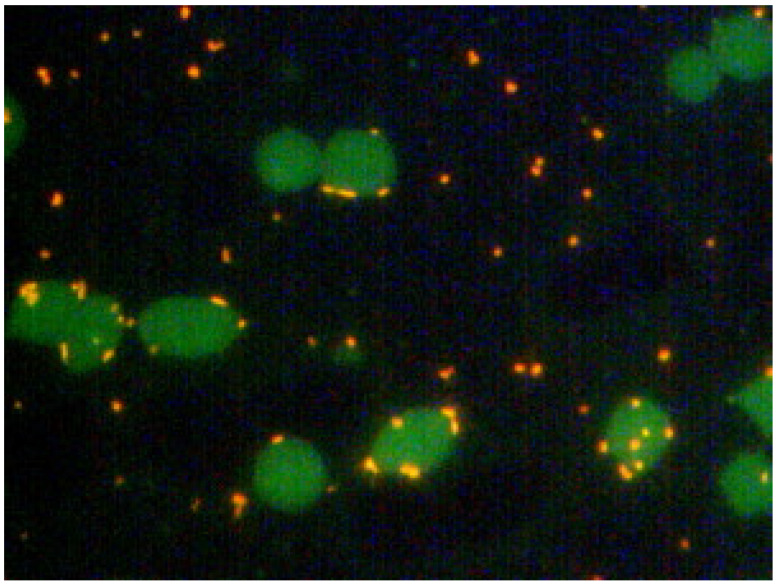
Acridine orange-stained blood smear of *M. suis*-infected pigs. *M. suis* cells are abundant and the staining of their DNA makes them appear as orange dots on the erythrocyte surface [50] (figure owned by the authors, license for re-use is granted by Elsevier, License number 5819149239986).

Additionally, fluorescence microscopy with acridine orange-stained blood smears was often used to detect *M. suis*, where the bacterial cells appear as light-orange dots with a yellow-green undertone [50,51]. An example of an acridine orange-stained blood smear from a pig infected with *M. suis* is shown in Figure 4 [50].

Microscopic methods have some disadvantages when it comes to sensitivity and specificity [51,105]. Dying artefacts as well as Howell–Jolly bodies can easily be confused with bacteria and, thus, can lead to a misinterpretation of examination results [127]. Moreover, microscopic detection only seems to be reliable with a certain bacterial load [51]. Since chronically infected animals usually have lower bacterial blood loads, false negative results can easily occur [51]. Furthermore, microscopy is not suitable for species differentiation of the porcine haemoplasmas.

### 6.3. Molecular Methods

Molecular detection methods are now commonly used for direct pathogen detection. Various conventional and real-time PCR protocols targeting different genes have been published and used for the detection of *M. suis*, *M. parvum* and ‘*Ca*. M. haemosuis’ in porcine blood samples. While most of the protocols are cross-specific for the haemoplasma species, others allow precise differentiation of the three porcine haemoplasma species without subsequent sequencing [19,36,37,38,82,100,127]. Table 6 provides an overview of published PCR protocols used for porcine haemoplasmas that allow species differentiation without further amplicon sequencing.

Apart from PCR protocols, single protocols for other molecular methods for direct pathogen detection, e.g., loop-mediated isothermal amplification (LAMP) or in situ hybridization, have been published [127,128].

### 6.4. Serological Methods

Protocols for the detection of antibodies against *M. suis*, but not for the detection of antibodies against *M. parvum* or ‘*Ca*. M. haemosuis’, have been described in the literature. However, according to available publications, serological methods do not seem to be used commonly in the diagnosis of *M. suis* infections in the field. The development and use of meaningful test systems require the availability of suitable test antigens. However, antigen availability is strongly limited due to the lack of in vitro cultivation. Antigens purified from the blood of *M. suis*-infected pigs have been used in enzyme-linked-immunosorbent assays (ELISAs), but their use is accompanied by some disadvantages, like standardization limitations, high variability in antigen structure and low availability due to the need for animal sampling [129,130,131]. Two recombinant *M. suis* antigens have been described and used in routine diagnostics in ELISAs [132,133]. For standard use of antibody detection to diagnose haemoplasma in pigs, the role of antibodies in IAP should be better investigated. In addition, possible cross-reactions with other haemoplasma species and non-haemotrophic mycoplasma in pigs should be thoroughly investigated. Only then can the suitability of antibody detection be assessed for practical use.

## 7. Prevention and Treatment

With a lack of commercial vaccines, no pathogen-specific preventive measures against porcine haemoplasmas are currently available. So far, only two vaccination trials for *M. suis* have been published. Whereas one experiment was able to induce an immunological response resembling partial protection against *M. suis* in piglets [134], another experiment demonstrated an exacerbation of clinical signs in vaccinated pigs after the challenge [135].

Considering the blood-dependent transmission route of haemoplasmas, combating biting and blood-sucking arthropods and one-use needle practices can help reduce transmission [133].

For all three porcine haemoplasma species, antibiotic treatment using tetracyclines has been considered successful in many cases to improve the clinical condition of infected animals as well as to reduce the number of bacteria in the blood [35,40,47,62,108]. Complete elimination of haemoplasmas through antibiotic treatment, however, was not achieved in all the cases [3]. On the contrary, antibiotic treatment was unsuccessful in the case of acute IAP induced by experimental infection in the study by Groebel et al. [44]. The use of antibiotics against subclinical and chronic infections, as they are commonly found in the field, has not yet been discussed. This represents a major knowledge gap for practitioners when supervising affected farms. As a supportive measure, oral administration of glucose helped to counteract hypoglycaemia during acute infectious anaemia caused by *M. suis* [40].

## 8. Conclusions

This review provides an overview of current knowledge on the three porcine haemoplasma species *M. suis, M. parvum* and ‘*Ca*. M. haemosuis’. This review also highlights gaps in our current knowledge, such as mechanisms of host–pathogen interactions and the significance of subclinical infections in naturally infected pigs. This evaluation of existing studies also illustrates the limited availability of data on ‘*Ca.* M. haemosuis and *M. parvum* compared with *M. suis*. Increasing our knowledge and understanding of all three porcine haemoplasmas may assist practitioners with recognizing haemoplasma-associated disease, interpreting diagnostic results and guiding clinically relevant decisions in prevention and treatment.

## Figures and Tables

**Table 1:** Porcine haemoplasma species.

Haemoplasma Species	Year of First Description	Reference
*Mycoplasma suis*, formerly *Eperythrozoon suis*	1950	[27]
*Mycoplasma parvum*, formerly *Eperythrozoon parvum*	1950	[27]
‘*Candidatus* Mycoplasma haemosuis’	2017	[30]

**Table 2:** Macroscopic and microscopic abnormalities described in *M. suis*-infected pigs.

Organ System	Macroscopic Observations	Microscopic Observations
Skin and mucous membranes	yellow, icteric discolourations [3,42,56,58]	
	paleness [55]	
Body cavities	yellow-stained fluid in cavities [58]	
Muscles	paleness [3]	
Lymphatic system	swelling and reddening (mediastinal and intestinal lymph nodes) [3]	blood resorption and increased phagocytosis of erythrocytes in lymph nodes [3]
		follicular hyperplasia (mesenteric lymph nodes) [3]
		sinus histiocytosis (mesenteric lymph node) [3]
		fibrin in sinuses of lymph nodes [3]
Vascular system	yellow discolouration of the tunica intima (Aorta) [3]	multifocal fibrin nets in medium-sized vessels [3]
		focal endothelial cell loss with thrombus formation, fibrin insudation and acute bleeding (Vena cava) [3]
		fibrin exudation (Aorta) [3]
		damages to endothelial surface, perforation of vessel walls (Aorta) [57]
		thrombi and fibrin networks close to endothelial lesions in the vessels
Bone marrow		erythrophagocytosis by macrophages [3]
Spleen	black discoloration [58]	hemosiderosis [59]
	enlarged in size [42,58]	
Lungs	hyperaemia of lung tissue [3]	alveolar and interstitial oedema [3]
	pulmonary oedema [3,58]	hyaline globules and thrombi of alveolar capillaries [3]
Liver	yellow-coloured herds, irregularly distributed [3]	hyaline thrombi of hepatic sinusoids and vessels [3]
	yellow discoloration [42,56,58]	dilatation of portal lymph vessels (liver) [3]
	paleness [3]	centrilobular necrosis [3,43,56]
	gall bladder oedema [3]	periportal necrosis [3]
		non-specific, reactive interstitial hepatitis [3]
		interlobular and intralobular hyperplasia [56]
		steatosis of intact hepatocytes [3]
Kidneys	hyperaemic tissue [3]	nonspecific inflammation of the connective tissue of the renal pelvis [3]
	blurred-whitish lesions [3]	non-purulent interstitial nephritis with dilatation of the lymph vessels [3]
	yellow discoloration [56]	lymphoid follicles (medulla and cortex) [56]
CNS		eosinophilic nerve cell necrosis (cortex) [3]
		acute bleeding and oedema (leptomeninx) [3]
		perivascular and histiocytic cell infiltrates in fourth ventricle [3]
		endothelial cell swelling (fourth ventricle) [3]

**Table 3:** *M. suis* proteins with confirmed or suspected functions as adhesion proteins.

*M. suis* Protein	Function in Adhesion to Erythrocytes	Function in Glucose Metabolism (Glycolysis)	Ref.
MSG1	confirmed	yes GAPDH (glyceraldehyde-3-phosphate dehydrogenase)	[65]
α-Enolase	confirmed	yes enolase function	[74]
OSGEP	confirmed	no	[75]
HspA1 (DnaK)	expected	no	[77]
*M. suis* hypothetical proteins (n = 7)	expected	unknown	[76]

**Table 4:** Reports describing the occurrence of porcine haemoplasmas worldwide.

Continent	Country	Reference
Asia	China	[16,30,84,85,86,87]
	Japan	[14,36]
	South Korea	[82,88]
	Thailand	[15]
Africa	Ghana	[89]
	Kenya	[83]
	Nigeria	[39,90,91]
	South Africa	[60]
South America	Argentina	[54,92,93]
	Brazil	[22,34,81,94,95,96,97]
North America	Canada	[98]
	United States	[25,26,31,46,99,100]
Europe	Austria	[101,102,103]
	Belgium	[104]
	France	[21,105]
	Germany	[20,35,37,38,51,106,107,108,109]
	Hungary	[110]
	Italy	[111]
	Portugal	[112]
	Serbia	[113,114]
	Switzerland	[106]
	The Netherlands	[80]
	United Kingdom	[43,61,115]

**Table 5:** Published prevalence data for *M. suis*, *M. parvum* and ‘*Ca*. M. haemosuis’.

	Country	Ref.	Category	Prevalence (Animal Level)		Prevalence (Herd Level)	
***M. suis***	Brazil	[97]	sows	18.2%	(22/121)	100%	(4/4)
			piglets	1.64%	(1/61)	25%	(1/4)
			boars	25.0%	(1/4)	25%	(1/4)
		[94]	slaughter pigs	76.2%	(112/147)	not available	
		[95]	sows + boars	54.7%	(35/64)	not available	
		[96]	sows	18.75%	(15/80)	40.6%	(11/27)
	France	[21]	sows	53.0%	(105/198)	100%	(10/10)
	Germany	[106]	piglets	10.6%	(17/160)	not available	
		[51]	feeder pigs	13.9%	(164/1176)	40.3%	(79/196)
		[37,38]	slaughter pigs	19.0%	(38/200)	50.0%	(10/20)
		[20]	sows	31.25%	(65/208)	76.2%	(16/21)
		[37]	sows	6.7%	(4/60)	not available	
		[20]	piglets	14.35%	(68/474)	not available	
		[37]	boars	0%	(0/0)	not available	
	Japan	[36]	feeder pigs + sows	5.0%	(6/120)	9.1%	(1/11)
	Korea	[82]	not specified	0.2%	(3/1867)	not available	
	Switzerland	[106]	sows	19.0%	(19/100)	not available	
***M. parvum***	Germany	[37]	sows	25.0%	(15/60)	not available	
		[37]	slaughter pigs	36.0%	(72/200)	not available	
		[37]	boars	4.4%	(8/183)	not available	
	Japan	[36]	feeder pigs + sows	15.0%	(18/120)	54.5%	(6/11)
	Korea	[82]	not specified	2.7%	(51/1867)	not available	
**‘*Ca*. M. haemosuis’**	Germany	[38]	piglets	4.5%	(28/622)	not available	
		[38]	sows	6.25%	(13/208)	14.28%	(3/21)
		[37,38]	slaughter pigs	17.5%	(35/200)	45.0%	(9/20)
		[37]	sows	21.7%	(13/60)	not available	
		[37]	boars	0%	(0/0)	not available	
	China	[30]	sows	36.0%	(31/86)	not available	
			fattening pigs	23.1%	(55/238)	not available	
	Korea	[82]	not specified	0.1%	(1/1876)	not available	

**Table 6:** PCR protocols that allow differentiation of the porcine haemoplasmas species.

Haemoplasma Species	Target Gene	Primer/Probe Name	Primer/Probe Sequence (5′ to 3′)	Reference
***M. suis***	msg1	Msg1-Fw (Forward)	ACAACTAATGCACTAGCTCCTATC	[106]
		Msg1-Rv (Reverse)	GCTCC TGTAGTTGTAGGAATAATTGA	
		Probe msg1-1 (Probe 1)	TTCACGCTTTCACTTCTGACCAAAGAC-fluorescein	
		Probe msg1-2 (Probe 2)	LCRed-640-CAAGACTCTCCTCACTCTGACCTAAGAAGAGC-phosphate	
	16S rRNA	M.s.713_for (Forward)	AACACCAGAGGCTAAGGCGA	[103]
		M.s.713_rev (Reverse)	TTACGGCGTGGACTACTGGG	
		MGB (Probe)	FAMTAATTGACGCTGAGGCTT	
	16S rRNA	RTsuisF (Forward)	CCCTGATTGTACTAATTGAATAAG	[100]
		RTsuisR (Reverse)	GCGAACACTTGTTAAGCAAG	
		MGBsuis2 (Probe)	FAM-TGRATACACAYTTCAG-MGBNFQ	
	16S rRNA	Suis 16S F (Forward)	AACGCATACTTAACTTT	[36]
		Suis 16S R (Reverse)	CAT ACT CCT ATT TAC CCG CT	
	23 S rRNA	MS_23SF1 (Forward)	GAAGTTTGAGCGAGAGCACAG	[127]
		MS_23SR3 (Reverse)	AGGGCTTAAGTTAGAAGCTTCAGC	
	23 S rRNA	MS_23SF1q (Forward)	TTGAAGTTTGAGCGAGAGCACAG	[127]
		MS_23SR1q (Reverse)	ACCCGTTGTCCATCAGTTACGT	
		MS_probe (Probe)	FAM-GTGAGAATCTTTCTAGCCGATTGATC-MGB-NFQ	
***M. parvum***	gap	MPaF (Forward)	ATGCTGGCGCTCCTAAAGTT	[37]
		MPaR (Reverse)	CTGCTGCAGCTCTAGCTCTT	
	16 S rRNA	Parvum 16S F (Forward)	AACACATATTTAACTTGCTC	[36]
		Parvum 16S R (Reverse)	CATATTCCTATTCATCCGCG	
**‘*Ca*. M. haemosuis’**	16 S rRNA	cmsf2 (Forward)	AAACTCTGATGGTACCTCCTGAATAAGTGA	[30]
		cmsr2 (Reverse)	CCTTCGCTGGGGATGTCAAACCT	
	gap	CMhsuisF (Forward)	TGCTTTGGCTCCTGTGGTTA	[38]
		CMhsuisR (Reverse)	GCAGCAGCACCTGTAGAAGTA	

## Data Availability

Not applicable.
